# Constraining classifiers in molecular analysis: invariance and robustness

**DOI:** 10.1098/rsif.2019.0612

**Published:** 2020-02-05

**Authors:** Ludwig Lausser, Robin Szekely, Attila Klimmek, Florian Schmid, Hans A. Kestler

**Affiliations:** 1 Institute of Medical Systems Biology, Ulm University, Ulm, Germany; 2 Leibniz Institute on Aging, Jena, Germany

**Keywords:** computational learning theory, invariances, classification, molecular profiles

## Abstract

Analysing molecular profiles requires the selection of classification models that can cope with the high dimensionality and variability of these data. Also, improper reference point choice and scaling pose additional challenges. Often model selection is somewhat guided by *ad hoc* simulations rather than by sophisticated considerations on the properties of a categorization model. Here, we derive and report four linked linear concept classes/models with distinct invariance properties for high-dimensional molecular classification. We can further show that these concept classes also form a half-order of complexity classes in terms of Vapnik–Chervonenkis dimensions, which also implies increased generalization abilities. We implemented support vector machines with these properties. Surprisingly, we were able to attain comparable or even superior generalization abilities to the standard linear one on the 27 investigated RNA-Seq and microarray datasets. Our results indicate that *a priori* chosen invariant models can replace *ad hoc* robustness analysis by interpretable and theoretically guaranteed properties in molecular categorization.

## 1. Introduction

Accurate and interpretable diagnostic models are a major ingredient in modern healthcare and a key component in personalized medicine [1,2]. They facilitate the identification of optimal therapies and individual treatments. These models are derived in long-lasting and cost-intensive data-driven processes, which are based on the analysis of high-dimensional marker profiles. In general, these search spaces exceed by far the possibility of manual inspection. Computer-aided systems are required for these screening procedures.

The canonical machine learning approach for deriving diagnostic classification models is the supervised learning scheme [3–5]. Here, a predictive model, a classifier, abstracts diagnostic classes from a set of labelled training examples.

Due to the data-driven nature of this learning process, the quality of a classifier is naturally dependent on the quality and amount of available samples. It can affect the generalizability and interpretability of a model. Both characteristics are of importance for the clinical setting. An incorrect prediction can lead to an incorrect treatment decision. A non-interpretable model is not verifiable and does not provide new insights in the molecular background of a disease. Small data collections might be supplemented by existing domain knowledge on the corresponding classification task or the recording process. It can provide information about hidden relationships or dependencies, which are too complex to be extracted from the data itself [6,7]. This information can structure the training process of a classification model, increasing both its accuracy and interpretability [8,9].

In the following, we focus on incorporating invariances into classification models [10]. Other approaches focus on regression applications [11,12]. That is the classification model and its predictions should not be affected by a specific data transformation. Typically, the terms *invariance* and *tolerance* are distinguished [13]. An invariant classifier completely neglects the influence of a data transformation; a tolerant one only reduces its influences. Invariances can be gained by model restrictions [14] or by initial data transformations [15,16]. They can also be enforced during the training process of a classifier [17–20]. For example, invariances can be learned by incorporating additional artificial samples in the training process of a classification model [21,22].

**Figure 1.**
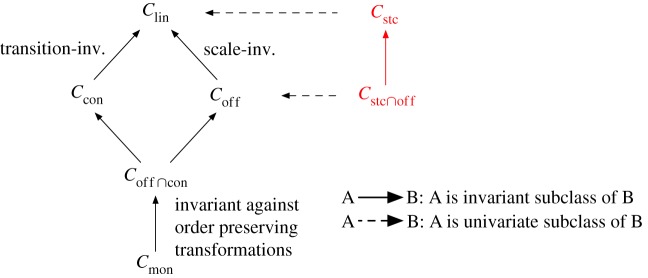
Invariant subclasses of linear classifiers. Linear classifiers $C_{\mathrm{lin}}$ can be organized in a hierarchy of four structural subgroups that imply different invariances. Each invariance counteracts the effects of a specific type of data transformation and preserves the predictions of the corresponding classification models. Some of these invariances can also be transferred to univariate predictors. This half-order is also reflected by a decrease in the Vapnik–Chervonenkis dimension from top to bottom, implying increased generalization ability. (Online version in colour.)

**Figure 2.**
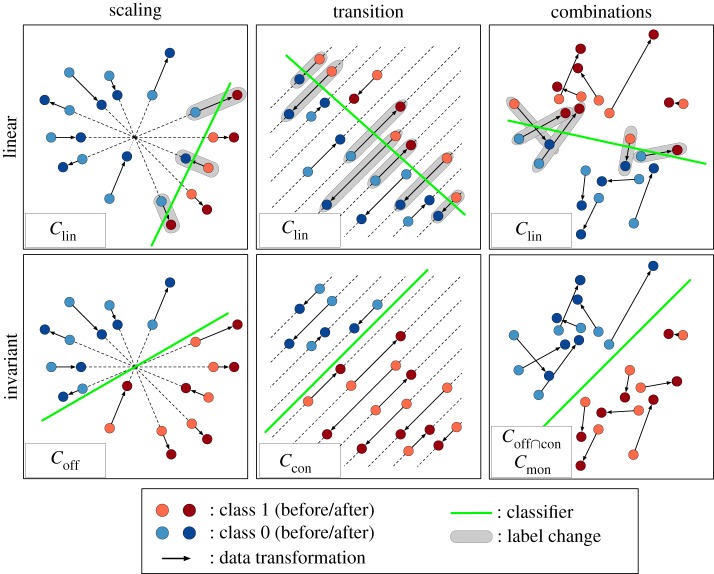
Structural properties of invariant linear classifiers: the first row gives examples of general linear classifiers $C_{\mathrm{lin}}$; the second row gives examples of the invariant concept classes $C_{\mathrm{off}}$, $C_{\mathrm{con}}$ and $C_{\mathrm{off}\cap \mathrm{con}}$ ($=C_{\mathrm{mon}}$ if $X=R^{2}$). Each column provides a dataset that is affected by a specific type of data transformation. From the left to the right, the datasets are affected by *global scaling*, *global transition* and the *combination* thereof. Data points that receive a different class label due to the data transformation are marked by a grey halo. (Online version in colour.)

Here, we impose invariance as a property of the underlying concept class of a classifier [23,24]. We generate four subclasses of linear classifiers that directly induce invariances to different data transformations (figure 1). Their structural characteristics are shown in figure 2 and listed in table 1. The theoretical properties of the concept classes and their implications on model complexity are elaborated in §2. The performance of invariant classifiers is evaluated in experiments with artificial datasets and gene expression profiles (§3). The corresponding results are shown in §4 and discussed in §5.

**Figure 3.**
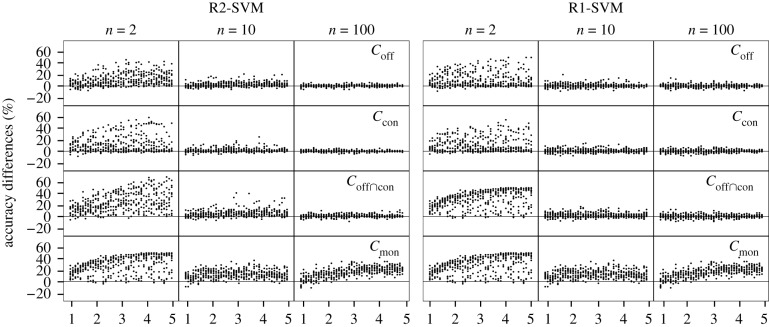
Evaluation of experiments on artificial datasets: the accuracy differences between SVM_lin_ and the invariant SVMs in noise-free experiments are shown. The rows show the different invariant classifiers. The columns provide the dimensionality of the underlying datasets *n* = {2, 10, 100}. The experiments are organized ascending according to the distances of the class centroids *d* (*x*-axis). The *y*-axis provides the accuracy difference. A positive value denotes a higher accuracy of the SVM_lin_. For each value of *d*, 10 experiments with different class centroids are shown.

**Table 1.** Overview in the discussed subclasses of linear classifiers. The concept classes are reported by their name, their structural properties, their invariances and their requirements on available measurements.

name structural properties	invariant to	required features ||**w**||_0_
(standard) linear classifier: $C_{\mathrm{lin}}=\{{𝟙}_{\left[\langle w,x\rangle \geq t\right]}\;|\;w\in R^{n},\;t\in R\}$	—	[1; *n*]
single threshold classifier: $C_{\mathrm{stc}}=\{{𝟙}_{\left[wx^{\left(i\right)}\geq t\right]}\;|\;w=\pm 1,i\in \{1,\ldots ,n\},t\in R\}$	—	1
offset-free linear classifier: $C_{\mathrm{off}}=\{{𝟙}_{\left[\langle w,x\rangle \geq 0\right]}\;|\;w\in R^{n}\}$	$f_{a}\;:\;x\mapsto a\cdot x$, with $a\in R^{+}$	[1; *n*]
offset-free single threshold classifier: $C_{\mathrm{stc}\cap \mathrm{off}}=\{{𝟙}_{\left[wx^{\left(i\right)}\geq 0\right]}\;|\;w=\pm 1,i\in \{1,\ldots ,n\}\}$	$f_{a}\;:\;x\mapsto a\cdot x$, with $a\in R^{+}$	1
linear contrast classifiers: $C_{\mathrm{con}}=\{{𝟙}_{\left[\langle w,x\rangle \geq t\right]}\;|\;\langle w,1\rangle =0,\;w\in R^{n},\;t\in R\}$	$f_{b}\;:\;x\mapsto x+b$, with b=b⋅1, b∈R	[2; *n*]
offset-free linear contrast classifier: $C_{\mathrm{off}\cap \mathrm{con}}=\{{𝟙}_{\left[\langle w,x\rangle \geq 0\right]}\;|\;\langle w,1\rangle =0,\;w\in R^{n}\}$	$f_{a,b}\;:\;x\mapsto ax+b$, with **b** = *b* · **1**, $a\in R^{+}$, b∈R	[2; *n*]
pairwise comparisions: $C_{\mathrm{mon}}=\{{𝟙}_{\left[x^{\left(i\right)}-x^{\left(j\right)}\geq 0\right]}\;|\;i\neq j,\;i,j\in \{1,\ldots ,n\}\}$	$f_{g}(x)\;:\;\left(\begin{array}{c} x^{\left(1\right)}\\ \vdots \\ x^{\left(n\right)} \end{array}\right)\mapsto \left(\begin{array}{c} g(x^{\left(1\right)})\\ \vdots \\ g(x^{\left(n\right)}) \end{array}\right),$ with $\forall x^{\left(i\right)},x^{\left(j\right)}\in R\;:\;g(x^{\left(i\right)})<g(x^{\left(j\right)})\;⟺\;x^{\left(i\right)}<x^{\left(j\right)}$	2

## 2. Material and methods

We use following notation throughout the article. A classifier will be seen as a function

2.1 c : X⟶Y,

mapping from the feature space X to the label space Y. The class label of a single sample x∈X is denoted by y∈Y. Most of the discussion will be focused on binary classification problems (e.g. Y={1,0}). We assume the feature space to be embedded in an *n*-dimensional Euclidian space $X\subseteq R^{n}$. A sample is represented as a vector **x** = (*x*^(1)^, …, *x*^(*n*)^)^*T*^.

The optimal structure of a classifier *c* is typically unknown *a priori*. It has to be learned in an initial training phase consisting of two major steps. First, a concept class C has to be chosen. It describes the structural properties and data-independent characteristics of a classifier.

In a second step, a classifier c∈C has to be adapted to the classification task. A training algorithm *l* has to be chosen that fits the classifier according to a set of labelled training examples $S_{\mathrm{tr}}={\left\{(x_{i},y_{i})\right\}}_{i=1}^{m}$,

2.2 $$l(S_{\mathrm{tr}},C)\mapsto c_{S_{\mathrm{tr}}}\in C.$$

We omit the subscript $S_{\mathrm{tr}}$ if the training set is known from the context.

The most important characteristic of a trained classifier is its generalization performance in predicting the class label of new unseen samples. It is typically estimated on an independent set of test samples $S_{\mathrm{te}}={\left\{(x_{i}^{\prime },y_{i}^{\prime })\right\}}_{i=1}^{m^{\prime }}$. A possible quality measure would be the classifiers empirical accuracy

2.3 $$A_{\mathrm{emp}}(c,S_{\mathrm{te}})=\frac{1}{\left|S_{\mathrm{te}}\right|}\sum_{(x,y)\in S_{\mathrm{te}}}{𝟙}_{\left[c(x)=y\right]}.$$

Here, ${𝟙}_{\left[p\right]}$ denotes the indicator function, which is equal to 1 if *p* is true and equal to 0 otherwise.

### 2.1. Invariant concept classes

Besides the overall generalization performance of a classifier, the invariances of its underlying concept class can be used for model selection. The predictions of the derived invariant classifiers will be unaffected by a family of data transformations [10]. For our analysis, we will use the following definition [14]:

**Definition 2.1.**

A classifier c : X→Y is called *invariant* against a parameterized class of data transformations $f_{\theta }\;:\;X\to X$ if

2.4 $$\forall \;\theta \in \Theta ,\forall \;x\in X:\;c(f_{\theta }(x))=c(x).$$

A concept class C is called invariant against *f*_*θ*_ if each c∈C is invariant against *f*_*θ*_.

Definition 2.1 calls a classifier invariant if its predictions are invariant against the influence of a parameterized class of data transformations. That is the classifier must be invariant against the influence of a data transformation for an unknown value of *θ* ∈ *Θ*. This implies that an invariant classifier is able to handle sample wise transformations. For a given test set $S_{\mathrm{te}}={\left\{(x_{i},y_{i})\right\}}_{i=1}^{m^{\prime }}$, an invariant classifier can neglect the effects of *m*′ distinct data transformations

2.5 $$\forall i\in \{1,\ldots ,m^{\prime }\}\;:\;c(f_{\theta_{i}}(x_{i}))=c(x_{i}).$$

A common parameter $\bar{\theta }$ that holds for all samples in $S_{\mathrm{te}}$ does not have to be estimated. A classifier invariant against *f*_*θ*_ is additionally invariant against sequences of data transformations

2.6 $$\forall \theta_{i},\theta_{\;j}\in \Theta \;:\;c(f_{\theta_{i}}(f_{\theta_{\;j}}(x)))=c(f_{\theta_{\;j}}(x))=c(x).$$

A concept class C that is invariant against *f*_*θ*_ summarizes all classifiers that share this invariance property. If this invariance can be traced back to a common structural characteristic of the classifiers the concept class can directly be used for training a classification model that is guaranteed to be invariant against *f*_*θ*_.

Here, we present structural subclasses of linear classifiers that directly lead to different invariances (table 1). Note that classifiers which constantly predict one particular class label (e.g. ∀x : c(x)=1 or ∀x : c(x)=0) are invariant against all possible data transformations $f_{\theta }\;:\;X⟶X$ but otherwise do not make any sense. Constant classifiers will, therefore, be excluded from the following analysis.

### 2.2. Linear classifiers

Linear classifiers separate the feature space via linear hyperplanes into two classes Y={0,1}. They are given by two parameters. The norm vector $w/∥w{∥}_{2},w\in R^{n}$ determines the direction of the hyperplane. The threshold t∈R can be seen as the distance from the hyperplane to the origin.

**Definition 2.2.**

The concept class of linear classifiers $C_{\mathrm{lin}}$ is given by

2.7 $$C_{\mathrm{lin}}=\{{𝟙}_{\left[\langle w,x\rangle \geq t\right]}\;|\;w\in R^{n},\;t\in R\}.$$

The concept class $C_{\mathrm{lin}}$ is one of the oldest ones for classification [25]. Its theoretical properties were, for example, analysed by Minsky & Papert [26] who demonstrated that Boolean functions exist that cannot be learned by linear classifiers (XOR problem). The flexibility of linear classifiers was first analysed by Cover [27]. It was proven that the probability of finding a linear classifier that perfectly separates a randomly labelled dataset increases with the dataset’s dimensionality.

Linear classification models are the underlying concept class for many popular training algorithms. For example, the perceptron [28], the linear discriminant analysis [25] and the support vector machine [29] were initially designed for linear classifiers. Although these training algorithms assume $C_{\mathrm{lin}}$ to be homogeneous, there exist different ways for separating the concept class into distinct subclasses. For example, linear classifiers can be distinguished by the number of features that are involved in their decision processes $∥w{∥}_{0}=\sum {\;}_{i=1}^{n}{𝟙}_{\left[w^{\left(i\right)}\neq 0\right]}$. Features that receive a weight of zero do not influence the decision process and can be omitted. The exclusion of noisy or meaningless features [30], the search for highly predictive markers [31] or the reduction of the model complexity [32] are possible reasons for a feature reduction to ||**w**||_0_ ≤ *k* < *n*.

Linear classifiers that rely on exactly one feature (||**w**||_0_ = 1) are summarized in the concept class of single threshold classifiers $C_{\mathrm{stc}}$ [33].

**Definition 2.3.**

The concept class of single threshold classifiers $C_{\mathrm{stc}}\subset C_{\mathrm{lin}}$ is defined as

2.8 $$C_{\mathrm{stc}}=\{{𝟙}_{\left[wx^{\left(i\right)}\geq t\right]}\;|\;w=\pm 1,i\in \{1,\ldots ,n\},t\in R\}.$$

These classifiers are typically used as base learners for classifier ensembles [33–35]. In this context, they are also called decision stumps or single rays. Single threshold classifiers are the only linear classifiers suitable for analysing single features independently.

Classifiers with ||**w**||_0_ = 0 are typically omitted. For technical reasons, we will treat a linear classifier with ||**w**||_0_ = 0 as a constant classifier (e.g. ∀x : c(x)=1 or ∀x : c(x)=0) in our analysis.

### 2.3. Invariant subclasses of linear classifiers

The following section provides an overview on the analysed invariant subclasses of linear classifiers. For each concept class, a theoretical proof on their invariance properties is given. An illustration of these concept classes can be found in figure 2. Their properties are summarized in table 1.

#### 2.3.1. Offset-free linear classifiers

The first invariant subclass of $C_{\mathrm{lin}}$ is the concept class of offset-free linear classifiers $C_{\mathrm{off}}$, which is characterized by fixing the threshold to *t* = 0.

**Definition 2.4 ($C_{off}$).**

The concept class of offset-free linear classifiers $C_{\mathrm{off}}\subset C_{\mathrm{lin}}$ is defined as

2.9 $$C_{\mathrm{off}}=\{{𝟙}_{\left[\langle w,x\rangle \geq 0\right]}\;|\;w\in R^{n}\}.$$

Fixing the threshold *t* = 0 forces the hyperplanes of offset-free linear classifiers through the origin, which leads to invariances different from those of general linear classifiers.

**Theorem 2.5.**

*A non-constant linear classifier* $c\in C_{\mathrm{lin}}$ *is invariant against* *global scaling*

2.10 $$f_{a}\;:\;x\mapsto a\cdot x,$$

*with* $a\in R^{+}$ *if and only if* $c\in C_{\mathrm{off}}$.

**Proof of Theorem 2.5.**

In order to prove the invariance of a linear classifier to a certain type of data transformation *f*_*θ*_, we have to prove that

2.11 $$\forall x\forall \theta \;:\;\langle w,f_{\theta }(x)\rangle \geq t\;⟺\;\langle w,x\rangle \geq t.$$

For global scaling, we get

2.12 ⟨w,a⋅x⟩≥t ⟺ a⋅⟨w,x⟩≥t

2.13 $$⟺\langle w,x\rangle \geq \frac{t}{a}.$$

For a general linear classifier $c\in C_{\mathrm{lin}}$ with *t* ≠ 0, there exists at least one $a\in R^{+}$ for which *t*/*a* ≠ *t* (e.g. *a* = |*t*|). For the case of *t* = 0, a linear classifier is offset-free $c\in C_{\mathrm{off}}$.  ▪

Omitting an offset (*t* = 0) makes a linear classifier invariant against the global scaling of test samples, while a standard linear classifier $c\in C_{\mathrm{lin}}$ might be misguided here.

Offset-free linear classifiers can be constructed independently of the number of involved features ||*w*||_0_ ≥ 1. In particular, single threshold classifiers can fulfil the structural property of $C_{\mathrm{off}}$.

**Definition 2.6 ($C_{stc\cap off}$).**

The concept class of offset-free single threshold classifiers $C_{\mathrm{stc}\cap \mathrm{off}}\subset C_{\mathrm{lin}}$ is defined as $C_{\mathrm{stc}}\cap C_{\mathrm{off}}$,

2.14 $$C_{\mathrm{stc}\cap \mathrm{off}}=\{{𝟙}_{\left[wx^{\left(i\right)}\geq 0\right]}\;|\;w=\pm 1,i\in \{1,\ldots ,n\}\}.$$

Although single threshold classifiers $c\in C_{\mathrm{stc}\cap \mathrm{off}}$ allow a scale-invariant classification according to single features, their applicability is limited due to the fixed threshold of *t* = 0. An alternative might be the usage of offset-free linear classifiers with ||*w*||_0_ = 2, which are, for example, used for constructing fold-change classifiers [36].

#### 2.3.2. Linear contrast classifiers

The second invariant subclass is the concept class of linear contrast classifiers $C_{\mathrm{con}}$ [14].

**Definition 2.7 ($C_{con}$).**

The concept class of linear contrast classifiers $C_{\mathrm{con}}\subset C_{\mathrm{lin}}$ is defined as

2.15 $$C_{\mathrm{con}}=\left\{{𝟙}_{\left[\langle w,x\rangle \geq t\right]}\;|\;\sum_{i=1}^{n}w^{\left(i\right)}=0,\;w\in R^{n},\;t\in R\right\}.$$

The norm vector of a linear contrast classifier is additionally constrained by $\sum_{i=1}^{n}w^{\left(i\right)}=0$. In the context of variation analysis, such linear mappings **w** are called contrasts [37,38]. The structural properties of a linear contrast classifier induce the invariance of $C_{\mathrm{con}}$.

**Theorem 2.8.**

*A non-constant linear classifier* $c\in C_{\mathrm{lin}}$ *is invariant against global transition*

2.16 $$f_{b}\;:\;x\mapsto x+b$$

*with* b∈R,b=b⋅1 *if and only if* $c\in C_{\mathrm{con}}$.

**Proof of Theorem 2.8.**

A global transition affects the decision of a linear classifier in the following way:

2.17 ⟨w,x+b⟩≥t ⟺ ⟨w,x⟩+⟨w,b⟩≥t

2.18 $$⟺\langle w,x\rangle +\sum_{i=1}^{n}w^{\left(i\right)}b\geq t$$

2.19 $$⟺\langle w,x\rangle +b\sum_{i=1}^{n}w^{\left(i\right)}\geq t$$

2.20 $$⟺\langle w,x\rangle \geq t-b\sum_{i=1}^{n}w^{(i).}$$

For a linear contrast classifier $c\in C_{\mathrm{con}}$ ($\sum_{i=1}^{n}w^{\left(i\right)}=0)$, the second term on the right-hand side is equal to zero. The scalar product is equivalent to 〈**w**, **x**〉 and the classification of the transformed sample is equivalent to the classification of the original sample.

For a general linear classifier $c\in C_{\mathrm{lin}}$ ($\sum_{i=1}^{n}w^{\left(i\right)}\neq 0)$, there exists a b∈R (e.g. *b* = 1) for which $d=b\sum_{i=1}^{n}w^{\left(i\right)}\neq 0$. This corresponds to a replacement of the original threshold *t* by *t* − *d* ≠ *t*.  ▪

The predictions of the linear contrast classifier $c\in C_{\mathrm{con}}$ are not affected by the individual transitions of the single samples while predictions of a general linear classifier $c\in C_{\mathrm{lin}}$ can be switched in both directions.

It is worth noting that there are no single threshold classifiers that can fulfil the additional constraint of $C_{\mathrm{con}}$. As a consequence, at least ||*w*||_0_ ≥ 2 features are needed for constructing a linear classifier that is invariant against global scaling. In the two-dimensional case ||**w**||_0_ = 2, the concept class is restricted to classifiers of type $c(x)={𝟙}_{\left[w^{\left(i\right)}x^{\left(i\right)}+w^{\left(j\right)}x^{\left(j\right)}\geq t\right]}$, *w*^(*i*)^ = −*w*^(*j*)^, *i* ≠ *j*, *i*, *j* ∈ {1, …, *n*}, t∈R.

#### 2.3.3. Offset-free contrast classifiers

The third invariant concept class consists of those linear classifiers that fulfil the constraints of both $C_{\mathrm{off}}$ and $C_{\mathrm{con}}$. It can be seen as the intersection of both concept classes.

**Definition 2.9 ($C_{off\cap con}$).**

The concept class of offset-free contrast classifiers $C_{\mathrm{off}\cap \mathrm{con}}\subset C_{\mathrm{lin}}$ is defined as $C_{\mathrm{con}}\cap C_{\mathrm{off}}$,

2.21 $$C_{\mathrm{off}\cap \mathrm{con}}=\left\{{𝟙}_{\left[\langle w,x\rangle \geq 0\right]}\;|\;\sum_{i=1}^{n}w^{\left(i\right)}=0,\;w\in R^{n}\right\}.$$

As a classifier $c\in C_{\mathrm{off}\cap \mathrm{con}}$ fulfils the structural properties of $C_{\mathrm{con}}$ and $C_{\mathrm{off}}$, it is invariant to both global scaling and global transition. In addition, it is invariant against combined effects.

**Theorem 2.10.**

*A non-constant linear classifier* $c\in C_{\mathrm{lin}}$ *is invariant against linear transformation that combine a global scaling and a global transition*

2.22 $$f_{a,b}\;:\;x\mapsto ax+b$$

*with* $a\in R^{+}$, b∈R,b=b⋅1 *if and only if* $c\in C_{\mathrm{off}\cap \mathrm{con}}$.

**Proof of Theorem 2.10.**

In case of linear transformations as described in equation (2.22), the decision of a linear classifier is influenced in the following way:

2.23 ⟨w,ax+b⟩≥t ⟺ ⟨w,ax⟩+⟨w,b⟩≥t

2.24 $$⟺\;a\langle w,x\rangle +\sum_{i=1}^{n}w^{\left(i\right)}b\geq t$$

2.25 $$⟺\langle w,x\rangle +\frac{b}{a}\sum_{i=1}^{n}w^{\left(i\right)}\geq \frac{t}{a}$$

2.26 $$⟺\langle w,x\rangle \geq \frac{t}{a}-\frac{b}{a}\sum_{i=1}^{n}w^{(i).}$$

For *a* = 1, the proof is now equivalent to the proof of theorem 2.8 for the invariance of $C_{\mathrm{con}}$. For all other $a\in R^{+}\setminus \{1\}$, the classifier is invariant if

2.27 $$t=\underset{:=d}{\underbrace{-\frac{b}{a-1}}}\sum_{i=1}^{n}w^{\left(i\right)},$$

where d∈R can be either positive or negative for different data transformations. The only unique threshold can be generated by forcing $\sum_{i=1}^{n}w^{\left(i\right)}=0$, which results in *t* = 0. The general linear classifier is, therefore, only invariant against *f*_*a*,*b*_, if $c\in C_{\mathrm{off}\cap \mathrm{con}}$.  ▪

As $C_{\mathrm{off}\cap \mathrm{con}}\subset C_{\mathrm{con}}$, the concept class again requires a minimal number of ||**w**||_0_ ≥ 2 features for constructing a non-constant classifier. For the two-dimensional case ||**w**||_0_ = 2, the concept class is restricted to classifiers of type $c(x)={𝟙}_{\left[w^{\left(i\right)}x^{\left(i\right)}+w^{\left(j\right)}x^{\left(j\right)}\geq 0\right]}$, *w*^(*i*)^ = −*w*^(*j*)^, *i* ≠ *j*, *i*, *j* ∈ {1, …, *n*}.

#### 2.3.4. The concept class of pairwise comparisons

We change the line of argumentation for introducing the fourth invariant concept class, which we call $C_{\mathrm{mon}}$. We first specify $C_{\mathrm{mon}}$ by its invariances and show afterwards that this subclass of linear classifiers can be defined by its structural properties.

**Definition 2.11 ($C_{mon}$).**

The concept class $C_{\mathrm{mon}}\subset C_{\mathrm{lin}}$ is defined as the subset of non-constant linear classifiers that is invariant against feature-wise strictly monotone increasing functions *f*_*g*_, where

2.28 $$f_{g}(x)\;:\;\left(\begin{array}{c} x^{\left(1\right)}\\ \vdots \\ x^{\left(n\right)} \end{array}\right)\mapsto \left(\begin{array}{c} g(x^{\left(1\right)})\\ \vdots \\ g(x^{\left(n\right)}) \end{array}\right),$$

and g : R→R fulfills

2.29 $$\forall x^{\left(i\right)},x^{\left(j\right)}\in R\;:\;g(x^{\left(i\right)})<g(x^{\left(j\right)})\;⟺\;x^{\left(i\right)}<x^{\left(j\right)}.$$

The concept class $C_{\mathrm{mon}}$ consists of linear classifiers that are invariant against all feature-wise strictly monotone increasing effects. This set of data transformations especially includes feature-wise nonlinear effects as, for example, strictly monotone polynomial or exponential transformations. The concept class $C_{\mathrm{mon}}$ is, therefore, at least as restrictive as $C_{\mathrm{off}\cap \mathrm{con}}$ and shares its invariance property with rank-based classifiers [15]. Theorem 2.12 states that $C_{\mathrm{mon}}$ is a real subset of $C_{\mathrm{off}\cap \mathrm{con}}$.

**Theorem 2.12.**

*The concept class* $C_{\mathrm{mon}}$ *is given by*

2.30 $$C_{\mathrm{mon}}=\{{𝟙}_{\left[w^{\left(i\right)}x^{\left(i\right)}+w^{\left(j\right)}x^{\left(j\right)}\geq 0\right]}\;|\;w^{\left(i\right)}=-w^{\left(j\right)},\;i\neq j,\;i,j\in \{1,\ldots ,n\}\}.$$

**Proof of Theorem 2.12.**

The proof of Theorem 2.12 is split into three parts. First, we show that no non-constant linear classifier $c\in C_{\mathrm{mon}}$ with ||**w**||_0_ = 1 exists. In a second step, we prove that the structural properties of a classifier $c\in C_{\mathrm{mon}}$ with ||**w**||_0_ = 2 match exactly the description given in equation (2.30). Finally, we prove that there is no non-constant classifier $c\in C_{\mathrm{mon}}$ with ||**w**||_0_ ≥ 3.

Case ||**w**||_0_ = 1: a linear classifier $c\in C_{\mathrm{mon}}$ has to be invariant to all feature-wise strictly monotone increasing functions *f*_*g*_. In particular, it has to be invariant to global scaling and global transition $C_{\mathrm{mon}}\subseteq C_{\mathrm{off}\cap \mathrm{con}}$. As there is no non-constant linear classifier $c\in C_{\mathrm{off}\cap \mathrm{con}}$ with ||**w**||_0_ = 1, there cannot be a non-constant linear classifier $c\in C_{\mathrm{mon}}$ with ||**w**||_0_ = 1.

Case ||**w**||_0_ = 2: the structural properties of $C_{\mathrm{off}\cap \mathrm{con}}⊇C_{\mathrm{mon}}$ for ||**w**||_0_ = 2 lead to the description of $C_{\mathrm{mon}}$ given in equation (2.30). The decision criterion can be rewritten as $c(x)={𝟙}_{\left[x^{\left(i\right)}\geq x^{\left(j\right)}\right]}$. As *g* is strictly monotone increasing

2.31 $$c(f_{g}(x))=\left\{ \begin{array}{cc} 1\; & \text{if}\;g(x^{\left(i\right)})>g(x^{\left(j\right)})⟺x^{\left(i\right)}>x^{\left(j\right)}\;\\ 1\; & \text{if}\;g(x^{\left(i\right)})=g(x^{\left(j\right)})⟺x^{\left(i\right)}=x^{\left(j\right)}\;\\ 0\; & \text{if}\;g(x^{\left(i\right)})<g(x^{\left(j\right)})⟺x^{\left(i\right)}<x^{\left(j\right)}\;\; \end{array},\right.\}$$

which corresponds to *c*(*f*_*g*_(**x**)) = *c*(**x**).

Case ||**w**||_0_ ≥ 3: for simplicity, we will omit feature dimensions that do not have any influence on the decision rule (*w*^(*i*)^ = 0). We will prove that for each linear classifier $c\in C_{\mathrm{off}}⊃C_{\mathrm{mon}}$ with ||**w**||_0_ = *n* ≥ 3 a sample $x\in R^{n}$ and a strictly monotone function *g* exist for which *c*(**x**) ≠ *c*(*f*_*g*_(**x**)). Without loss of generality, we will show that

2.32 $$\exists x\exists g\;:\;\sum_{i=1}^{n}w^{\left(i\right)}x^{\left(i\right)}\geq 0\;\text{and}\;\sum_{i=1}^{n}w^{\left(i\right)}g(x^{\left(i\right)})<0.$$

As ||**w**||_0_ = *n* ≥ 3, there are at least two weights which share the same sign. By permuting the ordering of the features, we can ensure that sign(*w*^(1)^) = sign(*w*^(*n*)^). We construct a sample $x\in R^{n}$ with

2.33 $$x^{\left(1\right)}<x^{\left(2\right)}=\cdots =x^{\left(n-1\right)}=0<x^{\left(n\right)}.$$

We furthermore construct a strictly monotone function *g* with *g*(0) = 0. This implies *g*(*x*^(1)^) < 0 and *g*(*x*^(*n*)^) > 0. The decision criterion in equation (2.32) can now be reduced to

2.34 $$\underset{>0}{\underbrace{-\frac{w^{\left(1\right)}}{w^{\left(n\right)}}x^{\left(1\right)}}}\leq x^{\left(n\right)}\;\text{and}\;\underset{>0}{\underbrace{-\frac{w^{\left(1\right)}}{w^{\left(n\right)}}g(x^{\left(1\right)})}}>g(x^{\left(n\right)}).$$

As *x*^(*n*)^ and *g*(*x*^(*n*)^) can be randomly chosen from $R^{+}$, we can find a pair of numbers that fulfil these equations. Similar proofs can be given for samples of class 0.  ▪

In contrast to the other invariant concept classes $C_{\mathrm{mon}}$ is directly coupled to a fixed number of features ||**w**||_0_ = 2. It is restricted to the unweighted pairwise comparison of two measurements *x*^(*i*)^ and *x*^(*j*)^. As a consequence, the training for a classifier $c\in C_{\mathrm{mon}}$ is directly coupled to a feature selection process for higher dimensional settings (*n* > 2). For a two-dimensional subspace, exactly two classification models exist (*w*^(*i*)^ = −*w*^(*j*)^, $w^{\left(i\right)}≶0$). They both share the same decision boundary.

#### 2.3.5. Vapnik–Chervonenkis dimension

Motivated by the need for invariance, we can further show that the identified subclasses also form a half-order of complexity classes which in turn can lead to an increased generalization ability. In general, the complexity of the invariant concept classes decreases with imposing additional invariances (figure 1). This, in turn, leads to a decrease in their susceptibility to overfitting [39].

The invariant concept classes can be seen as real subclasses of $C_{\mathrm{lin}}$. Here, we provide their Vapnik–Chervonenkis dimension (VCdim) as a combinatorial complexity measure [29] and show that they are lower than the VCdim of $C_{\mathrm{lin}}$. The VCdim is closely related to the probably approximately correct (PAC) learning framework [40], where it can be used to provide upper bounds on the generalization performance of a classifier. In the case of two classifiers with equal empirical performance, the classifier with the lower VCdim should be preferred [41].

A VCdim(C)=m gives the maximal number of arbitrarily chosen but fixed data points *m* that can be given all 2^*m*^ possible labellings when classified by members c∈C.

Our proofs are mainly based on the following theorem [29], where $X=R^{n}$:

**Theorem 2.13.**

*Let* *X* *be a finite-dimensional real vector space and let* *U* *be a finite-dimensional vector space of functions from* *X* *to* R.

*Let further*

V={v : X→{−1,1} : v(x)=sign (u(x)),u∈U,x∈X}.

*Then* VCdim(V)=dim(U).

**Proof.**

We follow the original proof here [29]: we first prove dim(U)≤VCdim(V) by showing that for all d≤dim(U), there are points **x**_1_, …, **x**_*d*_ such that for arbitrary labellings *y*_*i*_ ∈ { − 1, 1}, *i* = 1, …, *d* of these points, there is a function *u* ∈ *U* with *u*(**x**_*i*_) = *y*_*i*_.

Pick *d* linearly independent functions $u_{1},\ldots ,u_{d}\in U.$ Then, as these functions are linearly independent, there are points *x*_1_, …, *x*_*d*_ ∈ *X* such that the vectors

$$\left(\begin{array}{c} u_{1}(x_{1})\\ \vdots \\ u_{d}(x_{1}) \end{array}\right),\ldots ,\left(\begin{array}{c} u_{1}(x_{d})\\ \vdots \\ u_{d}(x_{d}) \end{array}\right)\in R^{d}$$

are linearly independent in $R^{d}.$ Therefore, their span is the whole $R^{d}$ and there are coefficients $a_{i}\in R$ with

$$y_{i}=\sum_{\;j=1}^{d}a_{j}u_{j}(x_{i}),\;i=1,\ldots ,d.$$

Setting $u(x)=\sum_{\;j=1}^{d}a_{j}u_{j}(x)\in U$ proves the claim.

We now prove VCdim(V)≤dim(U). Set k=dim(U)+1 and assume the contrary, namely VCdim(V)≥k.

Thus, for any set of labels *y*_*i*_ ∈ { − 1, 1}, there is a function v∈V,v(x)=sign(u(x)),u∈U and points **x**_*i*_ ∈ *X* such that

2.35 $$\mathrm{sign}(u(x_{i}))=y_{i},\;i=1,\ldots ,k.$$

For these points $x_{1},\ldots ,x_{k},$ define the vector space

2.36 $$\tilde{U}=\left\langle \{\left(\begin{array}{c} u(x_{1})\\ \vdots \\ u(x_{k}) \end{array}\right)\in R^{k}\;:\;u\in U\}\right\rangle \subset R^{k},$$

where 〈 · 〉 denotes the linear span. By assumption, $\dim(\tilde{U})\leq \dim(U)<k.$ Hence, there is a non-zero vector $a\in {\tilde{U}}^{⊥}$ in the orthogonal complement of $\tilde{U},$ i.e.

2.37 $$0=\sum_{i=1}^{k}a^{\left(i\right)}u(x_{i}),\;\;\mathrm{for}\;\mathrm{all}\;u\in U.$$

Then, by equation (2.35), there is a function *u* with $\mathrm{sign}\;u(x_{i})=\mathrm{sign}\;(a^{\left(i\right)}),i=1,\ldots ,k.$ Thus,

2.38 $$0=\sum_{i=1}^{k}a^{\left(i\right)}\mathrm{sign}\;(a^{\left(i\right)}).$$

As **a** ≠ **0**, we have a contradiction.  ▪

Using theorem 2.13, we are now able to provide the VCdim of the invariant concept classes of linear classifiers:

**Theorem 2.14.**

*Let* *n* *be the dimensionality of the input space* $X\subseteq R^{n}$. *The VC dimensions of the major concept classes given above* (table 1) *are*

(a) $\text{VCdim}\;(C_{\mathrm{lin}})=n+1.$
(b) $\text{VCdim}\;(C_{\mathrm{off}})=n.$
(c) $\text{VCdim}\;(C_{\mathrm{con}})=n.$
(d) $\text{VCdim}\;(C_{\mathrm{off}\cap \mathrm{con}})=n-1.$
(e) $\text{VCdim}\;(C_{\mathrm{mon}})\leq \max\{m|2^{m}\leq n(n-1)\}$.

**Proof of Theorem 2.14.**

In the proof, we make use of theorem 2.13, using a different vector space of functions *U* in every case.

(a) This result for general linear classifiers is well known in the literature [39].
(b) For the concept class $C_{\mathrm{off}}$, we chose the space of linear mappings $u\;:\;R^{n}\to R$ for *U*. It is well known that this space has dimension *n* [42]. Then theorem 2.13 implies the assertion.
(c) Consider the vector space $X={\left\langle (1,\ldots ,1)\right\rangle }^{⊥}$ which is the orthogonal complement of the space spanned by $(1,\ldots ,1)\in R^{n}$. Note that dim(X)=n−1 and there holds
  2.39 $$\sum_{i=1}^{n}w^{\left(i\right)}=0,\;w\in X.$$
  In theorem 2.13, we take for *U* the space of affine mappings from *X* to R [42], which has dimension (*n* − 1) + 1 = *n*.
(d) We argue exactly as in step (c), except that we take for *U* the space of linear mappings from *X* to R [42], which has dimension *n* − 1.
(e) For a fixed set of *m* samples $X={\left\{x_{k}\right\}}_{k=1}^{m}$ and fixed pair of feature dimensions *i* ≠ *j*, with $\forall k\;:\;x_{k}^{\left(i\right)}\neq x_{k}^{\left(j\right)}$ the classifiers in $C_{\mathrm{mon}}$ can result in at most two labellings
  $(a)\;y_{k}={𝟙}_{\left[x_{k}^{\left(i\right)}\geq x_{k}^{\left(j\right)}\right]}$
  $(b)\;{\bar{y}}_{k}={𝟙}_{\left[x_{k}^{\left(j\right)}\geq x_{k}^{\left(i\right)}\right]}={𝟙}_{\left[x_{k}^{\left(i\right)}<x_{k}^{\left(j\right)}\right]}=\neg {𝟙}_{\left[x_{k}^{\left(i\right)}\geq x_{k}^{\left(j\right)}\right]},$

which can be seen as a random labelling and its negation. In this way, $C_{\mathrm{mon}}$ can generate at most *n*(*n* − 1) distinct labellings in $R^{n}$. The set X can, therefore, receive all 2^*m*^ distinct labellings if 2^*m*^ ≤ *n*(*n* − 1). The maximal set size max{*m*|2^*m*^ ≤ *n*(*n* − 1)} is therefore an upper limit to $\text{VCdim}(C_{\mathrm{mon}})$.  ▪

### 2.4. Support vector machines

In the following, we consider (linear) support vector machines (SVMs) [29] as training algorithms for the invariant concept classes. SVMs are standard training algorithms for linear classifiers. In its original form, it is designed for maximizing the margin between the training samples and the hyperplane of a linear classifier. Several modifications of the original training algorithm exist [43]. For our experiments, we have chosen two *L*1 soft-margin SVMs.

#### 2.4.1. R2-support vector machines

The original SVM algorithm maximizes the margin by a regularization of the Euclidean norm ||**w**||_2_. It will be denoted as *R2-SVM* in the following. The training algorithm can be summarized by the following constrained optimization criterion:

2.40 $$\min_{w,t,\xi }\;\frac{1}{2}∥w{∥}_{2}^{2}+C\sum_{i=1}^{n}\xi_{i}$$

2.41 $$\text{s.t.}\;\forall i\;:\;y_{i}(w^{T}x_{i}-t)\geq 1-\xi_{i}$$

2.42 $$\;\forall i\;:\;\xi_{i}\geq 0.$$

In this context, we assume class labels Y={+1,−1}. The parameter *ξ*_*i*_ denotes the slack variables that enable the use of SVMs in the non-separable case by measuring deviation from the ideal condition. *C* is the cost parameter which induces a trade-off between margin maximization and minimization of the classification error.

#### 2.4.2. R1-support vector machines

A feature selecting version of the SVM replaces the regularization of the Euclidian norm by the regularization of the Manhattan norm ||**w**||_1_. We will use the term *R1-SVM* throughout the manuscript. The corresponding objective replaces equation (2.40) by

2.43 $$\min_{w,t,\xi }\;∥w{∥}_{1}+C\sum_{i=1}^{n}\xi_{i}.$$

The Manhattan norm is more sensitive to small weights near zero. The corresponding features will be removed from the linear decision boundary (*w*^(*i*)^ = 0).

#### 2.4.3. Training invariant support vector machines

The SVM training algorithm for linear classifiers can be restricted to invariant subclasses by additional constraints. These constraints reflect the structural properties of the subclasses.

2.44 $$\text{s.t.}\;t=0\;\text{if}\;c\in C_{\mathrm{off}}$$

2.45 $$\text{s.t.}\;\sum_{i=1}^{n}w^{\left(i\right)}=0\;\text{if}\;c\in C_{\mathrm{con}}$$

2.46 $$\;\;\text{s.t.}\;∥w{∥}_{0}=2\;\text{if}\;c\in C_{\mathrm{mon}}$$

The trained SVMs will be denoted as SVM_off_, SVM_con_, SVM${\;}_{\mathrm{off}\cap \mathrm{con}}$ and SVM_mon_. Note that a constraint has to be added for an invariant subclass and subclasses thereof. For example, if the SVM training algorithm should be applied to a classifier $c\in C_{\mathrm{off}\cap \mathrm{con}}$ both constraints for $C_{\mathrm{off}}$ and $C_{\mathrm{con}}$ have to be added.

## 3. Experiments

We have conducted experiments on artificial and real datasets in order to characterize how the choice of an invariant concept class influences the training of a linear SVM. All experiments were performed with help of the TunePareto software [44].

### 3.1. Experiments on artificial datasets

The performance of the invariant concept classes was examined in a sequence of controlled experiments on artificial datasets. A summary on all parameters is given in table 2. For these experiments, two normal distributions $N(c_{y},I)$, y∈Y were chosen as class wise distributions. Here, the class wise centroids are given by $c_{y}\in R^{n}$. The covariance of the classes is given by the identity matrix $I\in R^{n\times n}$. The centroid of the positive class $c_{1}={\left(c_{1}^{\left(1\right)},\ldots ,c_{1}^{\left(n\right)}\right)}^{T}$ is randomly selected according to a feature wise uniform distribution with $c_{1}^{\left(i\right)}∼U(0,10),i=1,\ldots ,n$. With that, it is ensured that the components of the centroid of the positive class are always positive. The centroid of the negative class is chosen in dependency of *c*_1_. Is is given by **c**_0_ = **c**_1_ + *d***w**/||**w**||_2_, where w∼N(0,1). In this way, the Euclidean distance between both centroids is ensured to be ||**c**_1_ − **c**_0_||_2_ = *d*.

**Table 2.** Summary of the analysed experiments on artificial datasets.

**experiments without noise**			
**tested classifiers:**			
concept classes:	$C\in \{C_{\mathrm{lin}},C_{\mathrm{off}},C_{\mathrm{con}},C_{\mathrm{off}\cap \mathrm{con}},C_{\mathrm{mon}}\}$		
training algorithms:	R2-SVM, R1-SVM		
**dataset parameters (varied):**		**dataset parameters (constant):**	
dimensionality:	*n* ∈ {2, 10, 100}	samples:	*m* = 2 × 50
distance of centroids:	*d* ∈ {1, 1.1, …, 5}		
repetitions:	*r* ∈ {1, …, 10}	**summary:**	
		number of experiments:	1 23 000
**experiments with noise**			
**tested classifiers:**			
concept classes:	$C\in \{C_{\mathrm{lin}},C_{\mathrm{off}},C_{\mathrm{con}},C_{\mathrm{off}\cap \mathrm{con}},C_{\mathrm{mon}}\}$		
training algorithms:	R2-SVM, R1-SVM		
**dataset parameters (varied):**		**random parameters (per sample):**	
experiment:	*ex* ∈ {*cl*., *sam*.}		
noise types:	*id* ∈ {1, …, 5}	$a∼U(10^{-5},p)$	
noise parameter:	*p* ∈ {0, …, 5}	$a∼U(10^{-5},p)$	
dimensionality:	*n* ∈ {2, 10, 100}	b∼U(−p,p)	
repetitions:	*r* ∈ {1, …, 10}	$c∼U(10^{-5},p)$	
**dataset parameters (constant):**		**summary:**	
samples:	*m* = 2 × 50	number of experiments:	18 000
distance of centroids:	*d* = 4		
**noise types (*id*)**			
1. none:	f : x↦x		
2. scaling:	$f_{a}\;:\;x\mapsto a\cdot x$		
3. transition:	$f_{b}\;:\;x\mapsto x+b$, with b=b⋅1, b∈R		
4. scaling and transition:	$f_{a,b}\;:\;x\mapsto a\cdot x+b$, with b=b⋅1, b∈R		
5. exponential:	$f_{c}\;:\;x\mapsto e^{0.2c\cdot x}$		

A single experiment is parameterized by the dimensionality of the feature vectors *n* ∈ {2, 10, 100} and the distance between the class centroids *d*. A set of 2 × 50 (two classes with 50 samples each) training samples was used for adapting the SVM classifiers and a set of 2 × 50 test samples was used for evaluating their accuracy. For each dimensionality *n* and distance *d*, the experiment was repeated for 10 different pairs of class centroids *r* ∈ {1, …, 10}.

#### 3.1.1. Experiments without noise

In this experiment, the training and test sets were analysed in their original form. The distance between the class centroids was varied *d* ∈ {1, 1.1, …, 5}. The performance of an invariant SVM is compared to its standard version. That means, an invariant R2-SVM is compared to the standard version of the R2-SVM and an invariant version of the R1-SVM is compared to the standard version of the R1-SVM.

#### 3.1.2. Experiments with noise

The artificial datasets were also used for experiments with different types of noise (table 2). For this purpose, the samples of a dataset were partially replaced by noisy copies. The influence of a noise type was regulated by a common noise parameter *p*. Experiments for six different noise levels were conducted ranging from *p* = 0 (no noise) to *p* = 5 (maximal noise). The distance between the class centroids was fixed to *d* = 4. Experiments were conducted for two different settings:

*Sample wise noise:* In this experiment, the individual samples of a test set $S_{te}$ were affected by individual noise effects *θ*_*i*_ ∈ *Θ* resulting in

3.1 $$S_{te}^{\prime }={\left\{(f_{\theta_{i}}(x_{i}^{\prime }),y_{i}^{\prime })\right\}}_{i=1}^{m^{\prime }}.$$

*Class wise noise:* Here, the samples of a pair of training and test sets $S_{tr}$, $S_{te}$ were affected by class wise noise effects. These effects were chosen individually for training and test samples *θ*_*y*_, *ψ*_*y*_ ∈ *Θ* resulting in

3.2 $$S_{tr}^{\prime }={\left\{(f_{\theta_{y_{i}}}(x_{i}),y_{i})\right\}}_{i=1}^{m},\;\text{and}\;S_{te}^{\prime }={\left\{(f_{\psi_{y_{i}^{\prime }}}(x_{i}^{\prime }),y_{i}^{\prime })\right\}}_{i=1}^{m^{\prime }}.$$

### 3.2. Experiments on transcriptome datasets

We have conducted experiments on 27 gene expression datasets, consisting of 22 microarray and five RNA-Seq datasets. A summary of the datasets is given in table 3. We used standard and established preprocessing methodologies for the transcriptome data [67]: RMA is used for gene expression measurements based on microarrays (luminescence measurements) and includes an internal log-transformation [68], for the count data from RNA-Seq experiments, we used RSEM which does not include an internal log-transformation [69,70].

**Table 3.** Summary of the used transcriptome microarray and RNA-Seq datasets. The classes, class wise sample sizes and number of features are shown.

id	tissue	class labels	samples	features
		(*y*_0_, *y*_1_)	(*m*_0_, *m*_1_)	(*n*)
*d*_1_:	bone marrow [45]	acute myeloid leukaemia (AML), mutated AML	21, 57	22 215
*d*_2_:	breast [46]	non-inflammatory, inflammatory	69, 26	22 215
*d*_3_:	bladder [47]	Ta, T1∪T2+	20, 20	7129
*d*_4_:	tongue [48]	normal mucosa, oral tongue squamous cell carcinoma	26, 31	12 558
*d*_5_:	soft tissue [49]	dedifferentiated liposarcoma, well-differentiated liposarcoma	40, 52	22 215
*d*_6_:	lymph node [50]	intermediate, monoclonal B-cell lymphocytosis	48, 44	22 215
*d*_7_:	brain [51]	healthy, schizophrenia	15, 13	12 558
*d*_8_:	kidney [52]	non-tumour kidney tissue, renal cell carcinoma (RCC)	23, 69	22 215
*d*_9_:	brain [53]	inbred alcohol-preferring, inbred alcohol-non-preferring	29, 30	8740
*d*_10_:	head and neck [54]	normal mucosa, head and neck squamous cell carcinoma	22, 22	12 558
*d*_11_:	lung [55]	normal tissue, adenocarcinoma	49, 58	22 215
*d*_12_:	lung [56]	adenocarcinoma, squamous cell carcinoma	14, 18	12 558
*d*_13_:	blood [57]	healthy, severe asthma	18, 17	32 321
*d*_14_:	blood [58]	diffuse large B-cell lymphoma, follicular lymphoma	19, 58	7129
*d*_15_:	prostate [59]	non-tumour prostate tissue, prostate tumour	50, 52	12 558
*d*_16_:	intestinal mucosa [60]	non-cystic fibrosis, cystic fibrosis	13, 16	22 215
*d*_17_:	fibroblasts [61]	healthy, macular degeneration	18, 18	12 558
*d*_18_:	prostate [62]	non-recurrent cancer, recurrent cancer	40,39	22 215
*d*_19_:	colon [63]	microsatellite instable tumour, microsatellite stable tumour	13, 38	7071
*d*_20_:	stomach [64]	non-cardia tumour tissue, cardia tumour tissue	72, 62	22 215
*d*_21_:	stomach [64]	normal gastric glands, tumour tissue	134, 134	22 215
*d*_22_:	skin [65]	melanoma, metastasis	25, 24	22 215
TCGA RNA-Seq [66]				
*d*_23_:	kidney	chrom. RCC (ChRCC), clear cell RCC (CCRCC)	91, 606	20 655
*d*_24_:	kidney	ChRCC, papillary RCC (PRCC)	91, 323	20 632
*d*_25_:	kidney	CCRCC, PRCC	606, 323	20 684
*d*_26_:	bile duct, pancreas	cholangiocarcinoma, pancreatic cancer	45, 183	20 439
*d*_27_:	liver, pancreas	HCC, pancreatic cancer	424, 183	20 657

As reference *k*-nearest neighbours classifiers [71] (*k*NN) with *k* ∈ {1, 3, 5}, random forests [72] (RF) with *nt* ∈ {100, 200, 300} trees and stacked auto-encoders [73] (SAE) with three layers of u,⌈u/4⌉,⌈u/16⌉ units and *u* ∈ {100, 500, 1000} were chosen.

All classifiers were evaluated in 10 × 10 cross-validations [3]. For this experiment, a dataset $S={\left\{(x_{i},y_{i})\right\}}_{i=1}^{m}$ is split into 10 folds of approximately equal size. Nine of them are combined to a training set $S_{tr}$ while the remaining one is used as a test set $S_{te}$ for evaluation. The procedure is repeated for 10 permutations of S.

## 4. Results

### 4.1. Results on artificial datasets

The results for the noise-free experiments on artificial datasets are shown in figure 3. The accuracy differences between SVM_lin_ and the invariant SVMs are given. A positive value denotes a higher accuracy of the SVM_lin_. In general, R2-SVMs and R1-SVMs react comparably on the test scenarios. It can be observed that the accuracy differences decrease with higher numbers of dimensions. Higher differences occur for larger distances of the class centroids. Over all R2-SVMs and R1-SVMs, both bias and variance decrease for increasing dimensionality. For *n* = 2, SVM_off_, SVM_con_, SVM${\;}_{\mathrm{con}\cap \mathrm{off}}$ achieve mean differences of 9.9% (IQR: [17.0%, 1.0%]), 10.1% (IQR: [17.0%, 1.0%]), 29.4%, (IQR: [42.0%, 18.0%]). For *n* = 100, they decrease to 0.2% (IQR: [1.0%, −1.0%]), 0.2% (IQR: [1.0%, −1.0%]), 0.2%, (IQR: [2.0%, −1.0%]).

**Figure 4.**
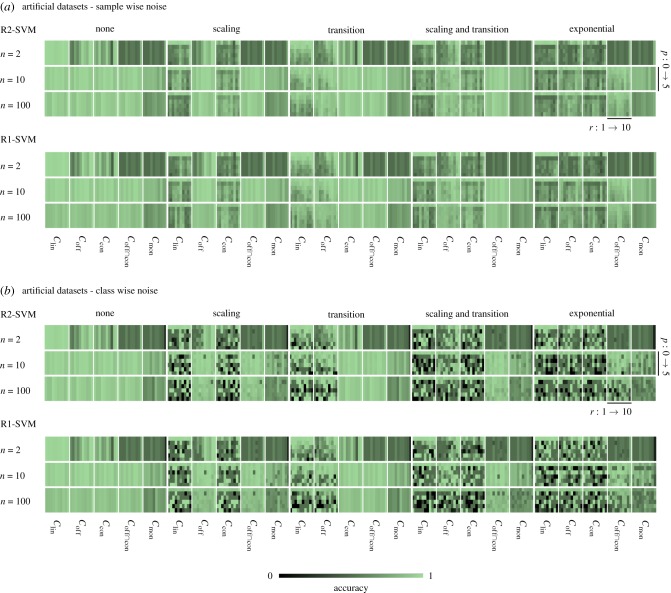
Accuracies achieved under the influence of data transformations: the figure provides the results of noise experiments with invariant R2-SVMs and R1-SVMs on artificial datasets. (*a*) The effects of sample wise data transformations on the test samples. (*b*) The influence of distinct class wise data transformations for training and test samples. The results are organized in blocks (from left to right), which correspond to the types of applied data transformations. Each column provides the results of a subclass of invariant classifiers. The rows give the dimensionality of the data *n* = {2, 10, 100}. Each box contains the result of 10 repetitions *r* ∈ {1, …, 10} and six increasing noise parameter *p* ∈ {0, …, 5}. (Online version in colour.)

The behaviour of the SVM_mon_ can be seen as an exception to these observations. Restricted to exactly two input dimensions, the SVM_mon_ cannot take advantage of the high-dimensional setting. Here, the bias and variance do not decline for higher dimensionality. For *n* = 2, a mean difference of 29.4% (IQR: [42.0%,18.0%]) can be observed. For *n* = 100, it achieves 14.9% (IQR: [21.0%,8.0%]).

The results of the noise experiments on artificial data are shown in figure 4. Figure 4*a* provides the results for the sample wise noise. In general, these experiments confirm the theoretical invariances against data transformations. It can be seen that for global scaling, SVM_off_, SVM${\;}_{\mathrm{off}\cap \mathrm{con}}$ and SVM_mon_ achieved equal accuracies for all noise levels. The performance of the SVM_lin_ variants of R2-SVM and R1-SVM drop rapidly. For the lowest noise level *p* = 1, mean accuracy losses of 34.6% (IQR: [40.5%, 33.8%]) are observed for the low-dimensional setting (*n* = 2) and 30.2% (IQR: [36.5%, 28.5%]) for the high-dimensional setting (*n* = 100). For global transition, the same invariant behaviour can be observed for the classifiers SVM_con_, SVM${\;}_{\mathrm{off}\cap \mathrm{con}}$ and SVM_mon_. Here, the lowest noise level *p* = 1 results in mean losses in accuracy of 2.4% (IQR: [4.0%, 0.0%]) for the SVM_lin_ variants in the low-dimensional setting (*n* = 2) and 4.6% (IQR: [6.0%, 0.8%]) for the high-dimensional setting (*n* = 100). The combination of global scaling and global transition resulted in equal accuracies for SVM${\;}_{\mathrm{off}\cap \mathrm{con}}$ and SVM_mon_ for every dimension and noise level. The SVM_lin_ variants showed mean accuracy differences of 34.7% (IQR: [42.3%, 31.0%]) in the low-dimensional setting (*n* = 2) and 29.6% (IQR: [35.8%, 30.0%]) in the high-dimensional setting (*n* = 100). After performing an exponential transformation on the test data, only SVM_mon_ led to equal accuracies for every dimension. The performance of the SVM_lin_ variants decreased by 44.8% (IQR: [48.0%, 45.8%]) in the low-dimensional setting (*n* = 2) and 38.6% (IQR: [43.3%, 38.0%]) in the high-dimensional setting (*n* = 100).

Figure 4*b* shows the results for the class wise noise. For global scaling, the SVM_off_ variants outperformed the SVM_lin_ variants in mean by 19.8% (IQR: [40.3%, 0.0%]) accuracy over all noise levels and all repetitions in the low-dimensional setting (*n* = 2). For the high-dimensional setting, a mean improvement of 35.3% (IQR: [48.3%, 3.0%]) accuracy was observed. For the global transition, the SVM_con_ gained in mean 8.1% (IQR: [27.3%, −12.5%]) accuracy for *n* = 2 and 33.5% (IQR: [47.3%, 0.0%]) for *n* = 100. In case of global scaling and transition, the SVM${\;}_{\mathrm{off}\cap \mathrm{con}}$ variants achieved in mean −2.8% (IQR: [11.0%, −24.8%]) less accuracy in the low-dimensional settings and 40.7% (IQR: [77.3%, 15.8%]) more accuracy in the high-dimensional setting. For the exponential transformation, the SVM_mon_ variants showed a performance decreased in mean by −14.5% (IQR: [0.0%, −40.3%]) for *n* = 2. It was in mean increased by 11.1% (IQR: [23.8%, −1.3%]) for *n* = 100.

### 4.2. Results on transcriptome datasets

**Figure 5.**
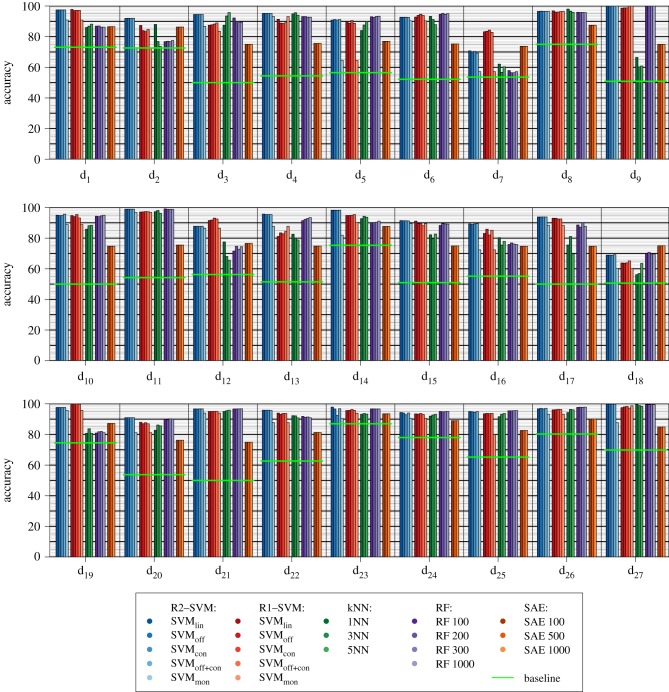
Results of 10 × 10 cross-validation experiments for transcriptome data: the mean accuracy is shown for the five concept classes of linear support vector machines (R2 and R1), for *k*NN with *k* ∈ {1, 3, 5}, for random forests with *nt* ∈ {100, 200, 300} trees and the stacked auto-encoders SAE with *u* ∈ {100, 500, 1000} units. Baseline denotes the performance of the classifier that always choses the larger class. (Online version in colour.)

The accuracies achieved on the microarray and RNA-Seq datasets are shown in figure 5 and tabularized in the electronic supplementary material.

The R2-SVM_lin_ outperformed the *k*NN (*k* ∈ {1, 3, 5}) on {25, 25, 26} datasets. It was inferior in {2, 2, 1} cases. The R1-SVM_lin_ was better than the *k*NN in {19, 21, 20} cases. In {7, 6, 7} settings the *k*NN was superior. In comparison to the RFs with *nt* ∈ {100, 200, 300, 1000} trees the R2-SVM_lin_ achieved better accuracies on {19, 20, 19, 18} datasets. Its accuracy was inferior on {7, 6, 6, 7} cases. The R1-SVM_lin_ outperformed the RFs on {12, 12, 12, 11} datasets. The RFs had higher accuracies on {15, 15, 15, 16} datasets. Th R2-SVM_lin_ showed better performance than SAE with *u* ∈ {100, 500, 1000} in {25, 25, 25} cases. They were outperformed on {2, 2, 2} datasets. For the R1-SVM, better performances were observed in {26, 26, 26} cases. Lower performance was gained on {1, 1, 1} datasets.

Overall, the respective invariant SVMs achieved better or equal results compared to the linear one in 41 of 54 cases. At the level of individual invariant linear SVMs, it can be observed that for 20 out of 27 datasets, an invariant R2-SVM was able to achieve the same or a higher mean accuracy than R2-SVM_lin_ (R1-SVMs: 21 datasets). R2-SVM_off_ outperformed R2-SVM_lin_ in four cases (R1-SVMs: 14 cases), achieved the same accuracy in 14 cases (R1-SVMs: two cases) and achieved a lower accuracy in nine cases (R1-SVMs: 11 cases). R2-SVM_con_ was able to achieve higher accuracies than R2-SVM_lin_ for 0 datasets (R1-SVMs: 18 datasets), equal accuracies on 17 datasets (R1-SVMs: 0 datasets) and lower accuracies for 10 datasets (R1-SVMs: nine datasets). R2-SVM${\;}_{\mathrm{off}\cap \mathrm{con}}$ was capable of achieving a higher accuracy than R2-SVM_lin_ in six cases (R1-SVMs: 14 cases), an equal accuracy in 12 out of 27 cases (R1-SVMs: 0 cases) and a lower accuracy in nine cases (R1-SVMs: 13 cases). The internally feature selecting R2-SVM_mon_ was never able to achieve a higher accuracy than R2-SVM_lin_, but the R1-SVM_mon_ outperformed its linear variant in four cases. For two (R1-SVM: 0) out of 27 datasets, R2-SVM_mon_ achieved the same accuracy as R2-SVM_lin_ and for 25 datasets (R1-SVM: 23 datasets) it led to a lower accuracy.

Besides the two-dimensional SVM_mon_ classifiers the R1-SVMs yields at the reduction of features that influence the final decision boundary. An overview on the mean percentage of used features is shown in the electronic supplementary material. In all experiments, no classifier selects more than 1% of the available features. The unconstrained SVM_lin_ constructed decision boundaries based on 0.06% to 0.51% of all features. The absolute mean size of these signatures lies in between 7.36 and 104.65 features. The invariant SVMs select comparable percentages of features. They lie in the ranges of 0.07% and 0.50% (SVM_off_), 0.07% and 0.86% (SVM_con_) and 0.07% and 0.51% (SVM${\;}_{\mathrm{off}\cap \mathrm{con}}$). This translates to a mean signature size of 9.93 and 102.76 (SVM_off_), 9.57 and 105.08 (SVM_con_) and 11.04 and 103.37 (SVM${\;}_{\mathrm{off}\cap \mathrm{con}}$).

## 5. Discussion

In this work, we derived four invariant types of linear classifiers. The structural properties of these models allow guaranteeing invariances in the presence of small collections of molecular profiles, where malicious variation might not even be detected.

From bench to bioinformatics, the extraction of molecular profiles requires multiple preprocessing steps which have to fulfil strict protocols and often need the collaboration of different experts or institutes. Deviations or differences of these protocols can lead to noise and bias, which might lead to imprecise estimates and wrong conclusions [38]. Invariances applied can be preventive in this context. A particular type of information, which is assumed to be affected, will be neglected in subsequent modelling processes. This work is related somehow to work by the group of Rainer Spang on zero-sum regression [11,12]; in fact, our classifier $C_{\mathrm{con}}$ corresponds to this concept class. Here, we extend and generalize this approach and also embed it into the PAC learning framework.

However, ignoring a specific type of information might result in diminished classification accuracies. Our experiments with invariant support vector machines indicate that incorporating invariances against global scaling and transition leads to approximately equal performance in high-dimensional biomarker settings. In this case, the differences in the complexity of the concept classes decrease. Decreased accuracies were only observed in experiments with low dimensionality. By contrast, restriction to exactly two input variables, which is required for the strictest invariant subclass, can affect a classifier’s performance.

Also, sparsity and invariance principles can be combined harmonically. The general findings described above can be observed for the feature selecting, invariant manhattan norm support vector machine. These results show that invariances can be incorporated into feature selection processes and might be used for constructing invariant marker signatures. In this case, the invariance on the full feature space is transferred to the reduced representation. The signatures of the invariant manhattan norm support vector machines have approximately the same length as their non-invariant counterpart. In our experiments, invariances against global scaling or transition result in signatures comprising in mean 0.07% to 0.51% of all available biomarkers, i.e. we obtain invariant signatures of mean length of 15.77 to 105.08 markers.

Our theoretical analysis, i.e. estimating the VC dimension of the four invariant concept classes, also reveals construction principles for other invariant concepts or more complex invariant classification models. The analysed hierarchy of concept classes does reflect not only an accumulation of invariances but also a reduction of the VC dimension. These analyses indicate that a restriction to invariant classification models also reduces the complexity of the corresponding concept classes and the risk of overfitting. Suitable models might be chosen according to the PAC learning framework.

Invariances can lead to constraints on the dimensionality of the input space of a linear classifier. While invariance against global scaling require multivariate profiles, the invariance against order-preserving functions is only guaranteed for the use of two covariates. Univariate linear classifiers do not match both criteria. These invariances do not, therefore, hold for architectures that are based on single-threshold classifiers. Among these architectures are standard implementations of hierarchical systems such as classification or regression trees or ensemble classifiers such as boosting ensembles. However, these systems can gain the desired invariances by completely replacing all univariate linear classifiers by higher dimensional invariant ones. Identifying suitable combinations of fusion architectures and invariant concept classes can be seen as a natural extension of this work.

## Supplementary Material

Read Me

## Supplementary Material

foldlists.tar

## Supplementary Material

invariantSVM_1.0.tar

## Supplementary Material

Results Accuracies

## Supplementary Material

ResultsFeaSelR1

## Data accessibility

Additional data can be found in the electronic supplementary material and under https://sysbio.uni-ulm.de/?Software:InvariantSVM.
